# Circulating catecholamines are associated with biobehavioral factors and anxiety symptoms in head and neck cancer patients

**DOI:** 10.1371/journal.pone.0202515

**Published:** 2018-08-20

**Authors:** Daniela B. Bastos, Bruna A. M. Sarafim-Silva, Maria Lúcia M. M. Sundefeld, Amanda A. Ribeiro, Juliana D. P. Brandão, Éder R. Biasoli, Glauco I. Miyahara, Dulce E. Casarini, Daniel G. Bernabé

**Affiliations:** 1 Psychosomatic Research Center and Oral Oncology Center, São Paulo State University (UNESP), School of Dentistry, Araçatuba, SP, Brazil; 2 Department of Medicine, Nephrology Division, Federal University of São Paulo (UNIFESP), São Paulo, Brazil; Technische Universitat Munchen, TranslaTUM, GERMANY

## Abstract

Studies have shown that stress-related catecholamines may affect cancer progression. However, little is known about catecholamine secretion profiles in head and neck cancer patients. The present study investigated plasma norepinephrine and epinephrine levels in head and neck squamous cell carcinoma (HNSCC) patients and patients with oral leukoplakia, as well as their association with clinicopathological and biobehavioral variables and anxiety symptoms. A total of 93 patients with HNSCC and 32 patients with oral leukoplakia were included. Plasma norepinephrine and epinephrine levels were measured by high performance liquid chromatography with electrochemical detection (HPLC-ED), and psychological anxiety levels were measured by the Beck Anxiety Inventory (BAI). Plasma norepinephrine and epinephrine concentrations were significantly higher in patients with oral and oropharyngeal squamous cell carcinoma (SCC) compared to non-cancer patients. Oral SCC patients displayed plasma norepinephrine levels about six times higher than oropharyngeal SCC patients, and nine times higher than oral leukoplakia patients (p < .001). Plasma epinephrine levels in oral SCC patients were higher compared to the oropharyngeal SCC (p = .0097) and leukoplakia (p < .0001) patients. Oropharyngeal SCC patients had higher plasma norepinephrine (p = .0382) and epinephrine levels (p = .045) than patients with oral leukoplakia. Multiple regression analyses showed that a history of high alcohol consumption was predictive for reduced plasma norepinephrine levels in the oral SCC group (p < .001). Anxiety symptom of “hand tremor” measured by the BAI was an independent predictor for higher plasma norepinephrine levels in HNSCC patients (β = 157.5, p = .0377), while the “heart pounding/racing” symptom was independently associated with higher plasma epinephrine levels in the oropharyngeal SCC group (β = 15.8, p = .0441). In oral leukoplakia patients, sleep deprivation and worse sleep quality were independent predictors for higher plasma norepinephrine levels, while severe tobacco consumption and higher anxiety levels were factors for higher plasma epinephrine levels. These findings suggest that head and neck cancer patients display sympathetic nervous system hyperactivity, and that changes in circulating catecholamines may be associated with alcohol consumption, as well as withdrawal-related anxiety symptoms.

## Introduction

Despite recent advances in cancer treatment, disease morbidity and psychological disorders continue affecting patient quality of life [1,2]. In general, many patients experience emotional and physical stress during the phases of cancer diagnosis, treatment, and post-treatment [3,4]. It is very common for oncological patients to display high levels of stress, anxiety, depression, and lack of social support [5–7]. This often results in neuroendocrine changes which may influence the progression of the tumor [8,9].

Central nervous system (CNS) perceptions of threat from environmental stressors, when experienced chronically, lead to downstream activation of neuroendocrine pathways, including the hypothalamic pituitary adrenal (HPA) axis and the autonomic nervous system (ANS) [3]. The HPA axis is governed by the hypothalamus and results in secretion of the hormone cortisol from the adrenals [9,10]. The sympathetic nervous system (SNS) is known for its role in the “fight-or-flight” stress response and secretes acetylcholine, which activates the secretion of catecholamines at nerve terminals by the adrenal medulla [3]. SNS activation, as well as subsequent release of catecholamines from sympathetic neurons and the adrenal medulla, mediate ANS stress responses [9]. The main catecholamines involved in the stress response are epinephrine (E) and norepinephrine (NE) [3,8]. Increased catecholamine levels have been found in individuals who experience acute or chronic stress, as well as mediate ANS influences on cardiac, respiratory, vascular, and other organ systems [8,9]. Investigations have indicated that higher catecholamine levels, derived from chronic stress and other emotional disorders like anxiety, may influence cancer progression [1,9,11]. Catecholamines may have modulatory effects on pathophysiological events which have a crucial role in cancer progression, including immunity impairment, angiogenesis, invasion, and modulation of inflammation [8,10,12,13]. For example, increased levels of NE and E can cause specific effects on the tumor-related immune response, including reduction of the number and activity of natural killer (NK) cells and dysregulation in the production of cytokines by lymphocytes [1,14,15]. Catecholamines derived from stress may induce increased levels of vascular endothelial growth factor (VEGF) and interleukin-6 (IL-6) on the tumor microenvironment, molecules which can drive angiogenesis and tumor growth [16–19]. Other consequences of increasing catecholamine levels in the tumor microenvironment may be the activation of molecules associated with increased cellular migration, as well as invasiveness (for example, metalloproteinases) of cancer cells and apoptosis inhibition mediated by anoikis [20,21].

Clinical studies have shown a direct association between catecholamine levels and psychological disorders in cancer patients. In a study with ovarian cancer patients, the authors showed a significant association between low subjective social support and higher intratumoral NE [11]. In other clinical investigations, plasma catecholamine levels were significantly correlated with anxiety scores in hepatocellular carcinoma patients, whereas the hormonal levels were associated with tumor differentiation [22]. Head and neck squamous cell carcinoma (HNSCC) is the 8th most common cancer worldwide, with oral and oropharyngeal tumors being its main subtypes [23]. There is a 5 to 25-fold increased risk of HNSCC in heavy smokers compared to non-smokers [23]. Alcohol consumption significantly increases the risk of cancer, especially in the upper aerodigestive tract, and a synergistic effect of smoking and high alcohol consumption is evident [23]. Patients with HNSCC can have several psychological disorders [24,25]. *In-vitro* investigations have shown that stress-related hormones can influence HNSCC cell behavior. In a previous study, we have found that elevated NE levels may increase oral squamous cell carcinoma (OSCC) cell proliferation through a pathway dependent on beta-adrenergic receptors [18].

However, there are almost no studies which have measured catecholamine systemic levels in HNSCC patients or their associations with psychological and clinicopathological variables. Xie *et al*. [26] found that circulating catecholamine levels were higher in oral cancer patients than patients with a benign oral tumor group, and were associated with depression and obsessive-compulsive symptoms. However, the histopathological diagnostics of the lesions which composed the oral cancer and oral benign tumor groups were not reported. In the present study, we measured the pre-treatment plasma NE and E levels in patients with oral and oropharyngeal cancer and oral leukoplakia, as well as their associations with clinicopathological and biobehavioral variables. We also examined the relationship between catecholamine levels and anxiety symptoms in these three groups.

## Patients and methods

### Ethics statement

This study was conducted according to the guidelines in the Declaration of Helsinki and approved by the Committee of Human Studies of the São Paulo State University (UNESP), School of Dentistry, Araçatuba, SP–Brazil (n°. 01314/2011). Review board requirements for written informed consent were waived because all personal identifying information was removed from the database prior to analysis.

### Patients

Patients over 18 years of age, with a primary tumor of oral or oropharyngeal SCC and oral leukoplakia (an oral benign lesion considered a potentially malignant disorder), were recruited at the Oral Oncology Center of São Paulo State University (UNESP) School of Dentistry in Araçatuba, Brazil, between 2012 and 2016. Inclusion criteria for the HNSCC groups were as follows: histopathological diagnosis of SCC without any previous treatment; primary tumor for the oral SCC group located in the anterior tongue, floor of the mouth, gingiva, lip, hard palate, buccal mucosa, or retromolar area; and primary tumor for the oropharyngeal SCC group located in the soft palate, uvula, tonsils, or base of tongue. Inclusion criteria for the leukoplakia group were clinical and histopathological diagnosis of oral leukoplakia according to the criteria of the World Health Organization (WHO) [27]. Patients with HNSCC who had concomitant tumors in another organ site were excluded. Exclusion criteria for all groups were past medical history of cancer, current pregnancy, inability to perform peripheral blood collection, and patients who did not consent to having the blood collected.

### Clinicopathological and biobehavioral variables

Clinical and histopathological information was obtained from medical records. The clinical parameters evaluated were as follows: age range (0–45, 45–65, and >65), gender, ethnicity, marital status, income, education, living with a relative, site of the primary tumor, clinical TNM classification, clinical staging, histopathological grade, pain occurrence related to primary tumor or lesion at the moment of the medical appointment, and type of treatment. The Charlson Comorbidity Index (CCI) was used to evaluate the comorbidity occurrence. The CCI is a widely used index and has been validated for head and neck cancer patients by Singh *et al*. [28] Information on health behaviors, such as tobacco and alcohol consumption, awareness of cancer diagnosis, and hours and quality of sleep, were obtained from medical records or provided by a patient health report. Patient sleep self-reports were obtained on the same day of blood collection, and information such as the number of hours of sleep the previous night and quality of sleep experience during the last night and last week was reported. Five subscales were used to describe the quality of sleep as follows: great, good, regular, very bad, and terrible.

### Anxiety levels and symptoms

Psychological assessments were obtained by interviews using the Beck Anxiety Inventory (BAI) on the same day that the blood sampling was done. The BAI is a 4-point, Likert-type scale, self-report inventory developed by Beck to measure the frequency of anxiety symptoms [29]. The total score of the inventory ranges from 0 to 63. Thirteen questions evaluate physiological symptoms, 5 questions evaluate comprehension, and 3 questions evaluate somatic and comprehension symptoms.

### Blood samples

All the patients were fasting on the morning of the blood collection, in order to prevent the effects of changes in oral intake on hormonal levels. Blood samples were collected from HNSCC and oral leukoplakia patients after diagnosis and before treatment. Samples were collected in the same controlled environment, with reduced outside stress stimuli, between 08:00 and 10:00 a.m. Peripheral blood was collected from the participants with a syringe treated with EDTA to prevent clotting. Subsequently, the bloods sample were centrifuged at 1500 rpm under refrigeration at 4°C for 20 minutes, and the plasma was then separated and stored at −80°C.

### Measurement of catecholamines

Plasma catecholamine (NE and E) levels were determined by high performance liquid chromatography using ion-pair reverse phase chromatography coupled with electrochemical detection (0.5 V) (HPLC-ED), as described by Naffah-Mazzacoratti *et al*. [30] and Di Marco *et al*. [31]. A plasma volume of 1 mL was added to 1 mL of Tris-buffer (pH 8.8, 2 moles/L) and 40 μl (8ng) of DHBA (internal standard, dihydroxybenzylamine), plus 50 mg of Al_2_O_5_. The suspension was vortex-mixed for 10 minutes and the precipitated alumina was washed three times. The catecholamines were eluted with 400 μl of perchloric acid after centrifugation at 1,500 g for 3 minutes. The supernatant was filtered and injected into the reverse phase column in the HPLC-ED. The NE and E concentrations were expressed as pg/mL.

### Statistical analysis

All data were stored in Microsoft Office Excel and the statistical analyses were performed by SAS software v.9.3. Normality was tested for continuous variables using the Shapiro-Wilk test. In case of normality, comparisons among groups were made using ANOVA followed by the multiple comparison Tukey test. Otherwise, comparisons were made using an adjustment for gamma distribution followed by the multiple comparison Wald test. The Chi-square and/or Fisher exact tests were used to evaluate associations between groups and categorical variables (clinicopathological, biobehavioral, and psychological). Correlations between the plasma NE and E levels in each group were tested using the Pearson correlation test. These analyses were performed separately in each group and also in a group composed of both oral and oropharyngeal SCC patients together (HNSCC group). Multivariate regression analysis was performed via the stepwise method of elimination, considering the levels of plasma NE and E as dependent variables, and the clinicopathological, biobehavioral, and psychological variables as explanatory. In addition, the median levels of plasma NE and E were considered, and a logistic multivariate model was fitted using a stepwise method for elimination, with the same explanatory variables. The results were presented in graphs as mean ± SE of mean (SEM) and a p value of < .05 was considered statistically significant.

## Results

### Epidemiologic and clinicopathological characteristics

One hundred and twenty-five patients met the inclusion criteria, of whom 71 had oral SCC, 22 had oropharyngeal SCC, and 32 had oral leukoplakia. The clinicopathological characteristics of the patients are described in Table 1. Participants were primarily male, white, and married or living with a partner. Mean ages were very similar for the oral SCC (57 years old), oropharyngeal SCC (54 years old), and leukoplakia patients (59 years old), and the majority of patients were between 45 to 65 years old (Table 1). The most common sites of the primary tumor for oral SCC patients were the tongue (36.6%) and the floor of the mouth (23.9%). For the oropharyngeal SCC group, the most common locations were the base of the tongue (50%) and the tonsils (22.7%). The groups did not differ significantly with respect to age, marital status, income, education, living with a relative, T classification, histopathological grade, or treatment. The leukoplakia group had a higher proportion of female patients (31.2%) compared to the oral SCC (12.7%) and oropharyngeal SCC groups (9.1%) (p = .0373). Although a higher proportion of non-white patients (33.8%) were in the oral SCC group compared to the oropharyngeal SCC (13.6%) and leukoplakia (6.3%) groups (p = .0188), the majority of the patients from the three groups had white ethnicity. The majority of patients from the three groups had at least one comorbidity. Leukoplakia (87.5%) and oropharyngeal (72.7%) SCC patients had higher scores of comorbidity measured by CCI compared to oral SCC patients (52.1%) (p = .0015). Oropharyngeal SCC patients showed higher regional metastasis occurrences (p = .001) and advanced disease stages (p = .0149) compared to oral SCC patients. As expected, HNSCC patients reported higher pain occurrence related to the primary tumor when compared to patients with oral leukoplakia (Table 1). Table 1 also shows information about treatment modalities used in oral and oropharyngeal SCC patients.

**Table 1:** Patient epidemiological and clinicopathological characteristics.

Variables	Leukoplakia 32 (%)	Oral SCC 71 (%)	Oropharyngeal SCC 22 (%)	p-Value
**Age (years)**				-
Mean (SD)	59.71 (8.81)	57.28 (11.69)	54.63 (7.17)	
**Age range (years)**				.2984
0–45 years-old	1 (3.1%)	7 (9.9%)	1 (4.5%)	
45–65 years-old	24 (75.0%)	46 (64.8%)	19 (86.4%)	
>65 years-old	7 (21.9%)	18 (25.3%)	2 (9.1%)	
**Gender**				**.0373**‡
Male	22 (68.8%)	62 (87.3%)	20 (90.9%)	
Female	10 (31.2%)	9 (12.7%)	2 (9.1%)	
**Ethnicity**				**.0188**‡
White	21 (65.6%)	46 (64.8%)	19 (86.4%)	
Non-white	2 (6.3%)	24 (33.8%)	3 (13.6%)	
Unknown/Missing data	9 (28.1%)	1 (1.4%)	0	
**Marital status**				.4956
Single	4 (12.5%)	20 (28.1%)	5 (22.7%)	
Married/living with a partner	19 (59.4%)	39 (55.0%)	14 (63.7%)	
Divorced/separated	6 (18.8%)	7 (9.9%)	1 (4.5%)	
Widowed	2 (6.2%)	5 (7.0%)	2 (9.1%)	
Unknown/Missing data	1 (3.1%)	0	0	
**Income (R$/month)**				.3764
<1.000,00	2 (11.1%)	3 (4.3%)	1 (4.8%)	
0,00–1.000,00	5 (27.8%)	21 (30.0%)	4 (19.0%)	
1.000,00–5.000,00	7 (38.9%)	14 (20.0%)	5 (23.8%)	
>5.000,00	4 (22.2%)	32 (45.7%)	11 (52.4%)	
**Education**				.7768
Illiterate	2 (6.2%)	3 (4.2%)	1 (4.5%)	
High school or less	16 (50.0%)	26 (36.6%)	10 (45.5%)	
College graduate	10 (31.3%)	38 (53.6%)	9 (41.0%)	
Postgraduate	1 (3.1%)	3 (4.2%)	1 (4.5%)	
Unknown/Missing data	3 (9.4%)	1 (1.4%)	1 (4.5%)	
**Live with some relative**				.4464
Yes	5 (15.6%)	9 (12.7%)	5 (22.7%)	
No	20 (62.5%)	62 (87.3%)	17 (77.3%)	
Unknown/Missing data	7 (21.9%)	0 (0%)	0 (0%)	
**CCI Score ¥**				**.0015**‡
0	4 (12.5%)	34 (47.9%)	6 (27.3%)	
1	12 (37.5%)	21 (29.6%)	11 (50.0%)	
2	4 (12.5%)	11 (15.5%)	4 (18.2%)	
3+	12 (37.5%)	5 (7.0%)	1 (4.5%)	
**T–Classification**				.8393
T1	-	17 (23.9%)	3 (13.7%)	
T2	-	21 (29.6%)	7 (31.8%)	
T3	-	16 (22.6%)	7 (31.8%)	
T4	-	17 (23.9%)	5 (22.7%)	
**Regional metastasis**				**.001**‡
N0	-	53 (74.7%)	8 (36.4%)	
N+	-	18 (25.3%)	14 (63.6%)	
**Clinical stage**				**.0149**‡
I	-	17 (23.9%)	1 (4.6%)	
II	-	20 (28.2%)	5 (22.7%)	
III	-	15 (21.1%)	2 (9.1%)	
IV	-	19 (26.8%)	14 (63.6%)	
**Histopathologic grade**				.8989
Grade I	-	11 (15.5%)	3 (13.6%)	
Grade II	-	42 (59.2%)	12 (54.5%)	
Grade III	-	2 (2.8%)	1 (4.6%)	
Unknown/Missing data	-	16 (22.5%)	6 (27.3%)	
**Pain**				**.0024**‡
With pain	4 (12.5%)	35 (49.3%)	8 (36.4%)	
No pain	27 (84.4%)	36 (50.7%)	13 (59.1%)	
Unknown/Missing data	1 (3.1%)	0 (0%)	1 (4.5%)	
**Treatment**				.1289
Surgery only	-	33 (46.5%)	3 (13.6%)	
Sur + RT	-	9 (12.7%)	2 (9.1%)	
RT	-	2 (2.8%)	1 (4.6%)	
Ch	-	9 (12.7%)	6 (27.3%)	
Ch + RT	-	9 (12.7%)	4 (18.2%)	
Sur + RT + Ch	**-**	4 (5.6%)	3 (13.6%)	
Other	**-**	5 (7.0%)	3 (13.6%)	

***Abbreviations*:** SCC, Squamous cell carcinoma; CCI, Charlson Comorbidity Index; Sr, Surgery; RT, radiotherapy; Ch, Chemotherapy.

**¥** CCI: Each numerical score equals different medical conditions, each weighted according to its potential to impact on mortality.

**‡** Values are considered statistically significant at p < .05

### Biobehavioral and psychological factors

The biobehavioral and psychological variables of the patients are presented in Table 2. Patients from the oropharyngeal SCC group consumed more tobacco and alcohol than the oral SCC (p = .0019) and leukoplakia patients (p = .0014). HNSCC patients showed worse sleep quality during the previous night than leukoplakia patients (p = .0464). In general, all patients showed lower anxiety levels measured by the BAI. About 87% of oral SCC, 85.7% of oropharyngeal SCC, and 75.9% of leukoplakia patients displayed minimum and light anxiety scores, while only 12.7% of oral SCC, 14.3% of oropharyngeal SCC, and 24.1% of leukoplakia patients reported moderate and severe anxiety scores. The total BAI mean scores did not differ significantly among the three groups (p = .3195).

**Table 2:** Patient biobehavioral and psychological characteristics.

Variables	Leukoplakia 32 (%)	Oral SCC 71 (%)	Oropharyngeal SCC 22 (%)	p-Value
**History of tobacco consumption €**				**.0019**‡
Non-smoker	5 (15.6%)	11 (15.5%)	1 (4.5%)	
Low	7 (21.9%)	31 (43.7%)	2 (9.1%)	
Moderate	3 (9.4%)	13 (18.3%)	6 (27.3%)	
Heavy	17 (53.1%)	16 (22.5%)	13 (59.1%)	
**History of alcohol consumption ᵼ**				**.0014**‡
Non-drinker	8 (25.0%)	10 (14.1%)	0 (0%)	
Low	9 (28.1%)	27 (38.0%)	4 (18.2%)	
Moderate	6 (18.8%)	16 (22.5%)	2 (9.1%)	
Heavy	9 (28.1%)	18 (25.4%)	16 (72.7%)	
**Sleep duration (Mean—hours)**	6.43	6.52	6.61	-
**Sleep quality (previous night)**				**.0464‡**
Great	12 (37.6%)	14 (19.7%)	4 (18.2%)	
Good	17 (53.1%	32 (45.1%)	10 (45.4%)	
Regular	2 (6.2%)	17 (23.9%)	2 (9.1%)	
Very bad	0 (0%)	5 (7.1%)	4 (18.2%)	
Terrible	1 (3.1%)	3 (4.2%)	2 (9.1%)	
**Awareness of cancer diagnosis**				.2249
Yes	-	38 (53.5%)	15 (68.2%)	
No	-	33 (46.5%)	7 (31.8%)	
**Beck Anxiety Inventory (BAI)**				.3195
Mean (SD)	11.55 (12.94)	8.35 (8.21)	10.95 (8.05)	
Minimum	20 (69.0%)	51 (71.8%)	12 (57.1%)	
Light	2 (6.9%)	11 (15.5%)	6 (28.6%)	
Moderate	5 (17.2%)	7 (9.9%)	3 (14.3%)	
Severe	2 (6.9%)	2 (2.8%)	0 (0%)	

***Abbreviations*:** SCC, Squamous cell carcinoma.

**€** Smoking: each category equals (per day) 10 cigarettes (manufactured, paper), two cigarettes (manufactured, straw), or two cigars or pipes of tobacco smoked.

**ᵼ** Alcoholism: each category equals (per day) two doses of distilled alcohol, two bottles of beer, or two glasses of wine consumed.

**‡** Values are considered statistically significant at p < .05

### Plasma norepinephrine and epinephrine concentrations

**Fig 1:**
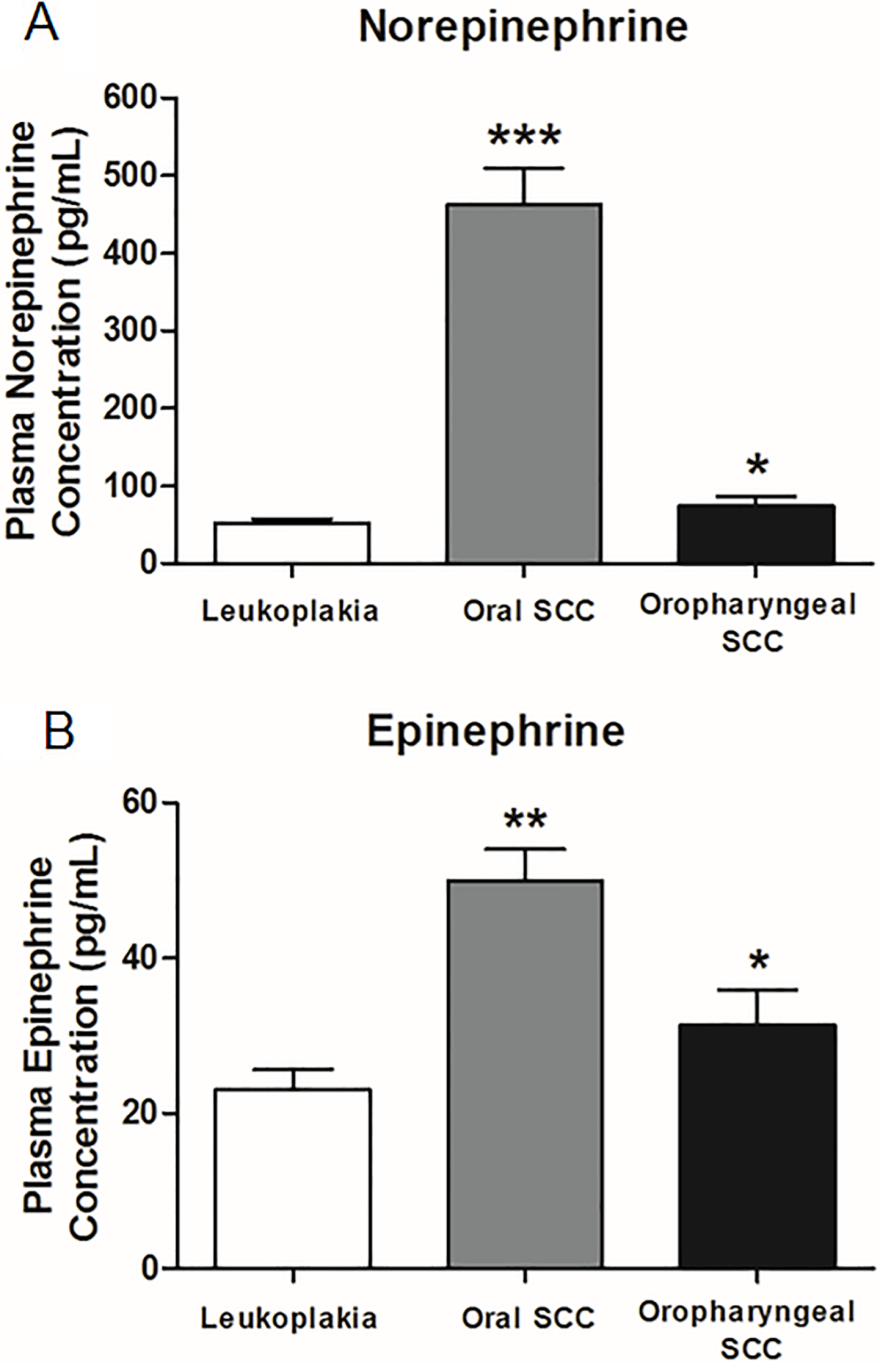
Plasma catecholamine concentrations. Plasma norepinephrine (A) and epinephrine (B) concentrations from oral leukoplakia (n = 32), oral SCC (n = 71) and oropharyngeal SCC patients (n = 22) were measured by HPLC. Results are expressed as mean ± SEM. ***p ≤.001: plasma NE levels from oral SCC group compared to oropharyngeal SCC and leukoplakia group; **p ≤.01: plasma E levels from oral SCC group compared to oropharyngeal SCC and leukoplakia group; *p ≤.05: plasma NE and E levels from oropharyngeal SCC group compared to leukoplakia group.

**Fig 2:**
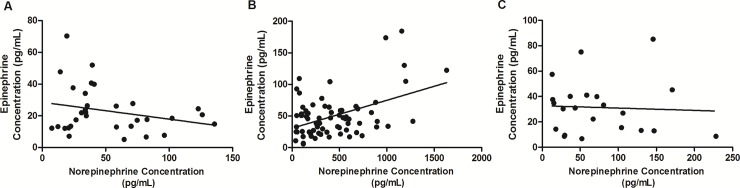
Correlation between plasma concentrations of norepinephrine and epinephrine. Correlations between plasma NE and E levels in oral leukoplakia (A), oral SCC (B) and oropharyngeal SCC patients (C) were analyzed using Pearson correlation test. Plasma NE levels were positively correlated with E levels in oral SCC patients (p = .0011).

The mean plasma NE concentration of oral SCC patients was approximately six times higher (462.03 *±* 47.53 pg/mL) than oropharyngeal SCC patients (74.46 *±* 12.52 pg/mL; p < .0001), and almost nine times higher than leukoplakia patients (51.69 *±* 6.28 pg/mL; *p* < .0001) (Fig 1A). Oropharyngeal patients showed higher plasma NE levels than those with leukoplakia (p = .0382) (Fig 1A). Plasma E levels in the oral SCC group were higher (49.94 *±* 4.10 pg/mL) compared to the oropharyngeal SCC (31.39 *±* 4.51 pg/mL; p = .0097) and leukoplakia (23.02 *±* 2.59 pg/mL; p < .0001) groups (Fig 1B). Oropharyngeal SCC patients had higher plasma E levels than patients with oral leukoplakia (p = .0452) (Fig 1B). Plasma NE levels correlated positively with E levels in oral SCC patients (p = .0011), but not in oropharyngeal SCC and leukoplakia patients (p>.05) (Fig 2).

### Associations between plasma catecholamine levels and clinicopathological variables

To investigate the association between plasma NE and E levels and clinicopathological variables, oral and oropharyngeal SCC patient samples were explored together (HNSCC group) and separately. Univariate analysis revealed no significant association between the plasma catecholamine levels and clinicopathological variables in the HNSCC group. However, HNSCC patients with advanced stages (stages III and IV) showed a trend towards lower levels of plasma NE (p = .0662). When HNSCC groups were analyzed separately, it was found that oral SCC patients who had a higher socioeconomic status showed higher plasma NE levels (p = .0217). Oropharyngeal SCC patients who were white and had a smaller tumor (T1 and T2) showed higher E levels compared to non-white patients (p = .0389) and to those with larger tumors (T3 and T4) (p = .0156). Based on the CCI score, univariate analysis showed that leukoplakia patients who had no occurrence of comorbidities had higher plasma E levels than patients with one or more comorbidities (p = .0031). Contrary to the findings in the oral SCC group, leukoplakia patients with a poor socioeconomic status had higher E levels compared to patients with better financial conditions (p = .0379). Table 3 shows data from the multivariate analysis. When the oral and oropharyngeal groups were analyzed together (HNSCC group), multivariate analysis showed no significant association between the plasma NE and E and the clinicopathological variables. Analyzing the groups separately, plasma catecholamines also were unrelated to any clinicopathological variables. Multivariate regression analysis showed that living with a relative (β = -4.44, p = .0015) was an independent variable associated with higher plasma E levels in the leukoplakia group (Table 3).

**Table 3:** Multiple regression coefficients of biobehavioral and psychological variables and plasma catecholamine measurements for leukoplakia group.

Variables	Leukoplakia		
	β	SE	p-Value
**Plasma Norepinephrine**			
Sleep Duration	-9.70	1.78	.0029
Sleep Quality	69.53	9.66	.0008
**Plasma Epinephrine**			
Living with a relative	-4.44	0.57	.0015
Tobacco consumption	1.54	0.27	.0051
BAI Score	7.16	0.61	.0003

***Abbreviations*:** *SE*, Standard error; BAI, Beck Anxiety Inventory. All values are considered statistically significant at p < .05

**β**: Parameter estimate.

### Associations among plasma catecholamine levels and biobehavioral variables and anxiety status

The results showed that HNSCC patients who had a history of higher tobacco (p = .0135) and alcohol consumption (p = .001) showed lower plasma NE levels compared to patients who consumed these addictive substances in lower intensity. In univariate analysis, global anxiety scores measured by the BAI symptom were not significantly associated with plasma NE and E levels in any of the groups. In addition, the present study investigated the association of each BAI subscale with the NE and E levels. The results showed that in the HNSCC group, NE levels were associated to the greater intensity of the “breathing difficulty” symptom, as measured by the BAI (p = .0366). When analyzing the groups separately, positive associations were also observed between this symptomatology with NE levels in oral SCC patients (p = .0383) and with E levels in oropharyngeal SCC patients (p = .026). No association was observed between these symptoms and catecholamine levels in leukoplakia patients. Table 4 shows the data from the multivariate analysis. Multivariate analysis showed that higher alcohol consumption was an independent predictor for lower NE levels (β = -171.7, p = .0002) in the HNSCC group. A similar result was found when the oral SCC group was analyzed separately. Moderate and severe oral SCC drinkers displayed lower plasma NE than light drinkers (β = -119.2, p = .0296) (Table 4). For the leukoplakia group, multiple regression model analysis showed that fewer hours of sleep (β = -9.70, p = .0029) and worse sleep quality the previous night (β = 69.53, p = .0008) were independent predictors for higher plasma NE, (Table 3). Severe tobacco consumption (β = 1.54, p = .0051) and higher anxiety levels (β = 7.16, p = .0003) were independent predictors for higher plasma E levels in leukoplakia patients (Table 3). When separately analyzing each subscale symptom measured by the BAI, the results showed that the “hand tremor” symptom (β = 157.5, p = .0377) in HNSCC patients was an independent predictor for higher plasma NE levels (Table 4). In oropharyngeal SCC patients, the “heart pounding/racing” symptom independently predicted higher plasma E levels (β = 15.8, p = .0441) (Table 4).

**Table 4:** Multiple regression coefficients of patient biobehavioral and psychological variables and plasma catecholamine levels for cancer groups.

Variables	HNSCC			Oral SCC			Oropharyngeal SCC		
	β	SE	p-Value	β	SE	p-Value	β	SE	p-Value
**Plasma Norepinephrine**									
Alcohol consumption	-171.7	44.1	.0002	-119.2	53.2	.0296	-	-	-
**Hand tremor**	157.5	74.2	.0377	-	-	-	-	-	-
**Plasma** **Epinephrine**									
Heart pounding / Racing	-	-	-	-	-	-	15.8	6.9	.0441

***Abbreviations*:** *SE*, Standard error; HNSCC, Head and Neck Squamous Cell Carcinoma; SCC, Squamous Cell Carcinoma. All values are considered statistically significant at p < .05

**β**: Parameter estimate

## Discussion

A growing number of clinical and pre-clinical studies have demonstrated that hormonal alterations resulting from chronic stress and other behavioral conditions may influence cancer progression [8,19,32]. Among the stress-related hormones, catecholamines such as NE and E have played a central role in the relationship between stress-related biological events and cancer progression [9]. However, few clinical studies have explored the catecholamine levels in cancer patients and their impact on the disease prognosis, or which variables would affect hormonal secretion. The results of the present study showed that patients with oral and oropharyngeal SCC exhibited plasma catecholamine (NE and E) levels significantly higher than leukoplakia patients. An interesting finding was that the highest difference peak was observed for NE concentrations in oral SCC patients. These patients displayed hormonal levels nine times higher than patients with oral leukoplakia, a benign but potentially malignant lesion, whose risk factor is similar to HNSCC. Some other investigations have reported increased rates of systemic catecholamine in cancer patients compared to individuals without cancer. Drott *et al*. [33] showed that urine excretion of E was significantly increased in a small sample of cancer patients suffering from malnutrition, when compared with equally malnourished control patients. Similar to the findings of the present study, Xie *et al*. [26] also found that oral cancer patients had higher plasma NE and E levels compared to patients with an oral benign tumor. However, in that study, only forty oral cancer patients were included and the authors did not specify which were the clinical and histopathological diagnoses of lesions made up of the oral benign tumor and cancer groups. On the other hand, a study of breast cancer patients [34] and another study of patients with different types of cancer [35] could not find differences in urinary catecholamine levels when cancer patients were compared to those observed in health controls. Surprisingly, the results of the present study showed that the mean concentration of plasma catecholamine in oral SCC patients was higher than that found in oropharyngeal SCC patients. This difference was also more expressive for plasma NE, whose levels in oral SCC patients were approximately six times higher compared to oropharyngeal SCC patients. It is not easy to explain the hormonal differences found between the two HNSCC groups. In a way, oral and oropharyngeal tumors exhibit relatively similar characteristics, including common risk factors such as smoking and alcoholism. In the present study, there were no significant differences between the two cancer groups for most of the clinicopathological and biobehavioral variables. However, oropharyngeal SCC patients displayed a higher occurrence of regional metastasis at the time of diagnosis and a history of increased consumption of tobacco and alcohol. Although the findings of the present study have shown patient’s history of increased alcohol consumption as the main independent variable associated with lower NE levels in HNSCC patients, it is presumptuous to point out this variable as the only one responsible for the lower NE levels observed in oropharyngeal cancer patients when compared to oral cancer patients. Due to the small sample of oropharyngeal SCC patients, other studies with a larger sample which explore other influencing variables are required.

When the multiple analysis to evaluate which variables had a predictive value to influence plasma catecholamine levels was performed, the results of the present study showed that higher alcohol consumption was an independent factor for lower plasma NE levels in the HNSCC group and the single oral SCC group. Moderate and severe drinkers displayed lower plasma NE than light drinkers. Few studies have explored the association between alcohol consumption and catecholamine levels in cancer patients. In two of these studies [11,26], one with ovarian cancer patients and another with oral cancer patients, no significant associations were found between alcohol consumption and plasma NE and E levels, although neither study reported a detailed alcohol consumption profile. Parlesak *et al*. [36] found that patients with alcoholic liver disease had increased urinary and plasma NE and E compared to healthy patients. In an experimental study, Kovács *et al*. [37] observed that ethanol administration in animals promotes a prompt increase in plasma NE and E, and hormonal levels also tend to increase during the tolerance/dependence phase. In the present study, blood collections from HNSCC patients were performed before or shortly after the disease diagnosis. Information regarding alcohol consumption was obtained from the medical records. This information reports the history of alcohol consumption throughout the life of the patient, but it does not take into account the last days or weeks before blood collection. Therefore, it cannot be ruled out that the patients may have been consuming alcohol in a different manner from what they reported in the medical interview in the days prior to the blood collection. We hypothesized that due to tumor-related symptoms, alcohol and tobacco consumption may decrease or even cease. Therefore, it is reasonable to believe that some of the alcohol-dependent patients in the present study could be in acute or chronic abstinence from alcoholism, which would have a direct impact on catecholamine secretion. Studies have reported evidence that abstinent alcohol-dependent patients can display a significant reduction in the plasma levels of NE or its metabolites, compared to actively drinking individuals [38,39]. These findings reinforce the importance of cross-sectional studies of cancer patients using retrospective data, and updating their clinicopathological and biobehavioral data up to the time of blood collection.

In the present study, univariate analysis showed that a history of higher tobacco consumption and advanced clinical staging were associated with lower NE levels in HNSCC patients, although these results were not maintained as independent variables in the multiple regression model. Lindell *et al*. [40] found that plasma concentrations of NE decreased during the smoking of a single cigarette, whereas those of E increased on smoking days. However, in general, clinical studies with non-cancer smokers have demonstrated that smoking leads to increased systemic levels of NE and E, which are strongly reduced after smoking withdrawal [41–43]. In a previous study, oral SCC patients were found to have increased plasma and salivary levels of cortisol, a stress-related hormone, compared to controls, and the hormonal levels were associated with advanced clinical staging [44]. In the present study, using a sample of oral cancer patients with a similar clinical profile to the aforementioned study, advanced clinical staging tended to be correlated with lower NE levels. These findings may suggest a different endocrine response for cortisol and NE in HNSCC patients with advanced clinical staging. Two different studies of ovarian cancer patients found no significant association between the disease stage and the plasma catecholamine levels [11,45]. However, Xie *et al*. [26], studying patients with oral cancer, and Liu *et al*. [22], studying patients with hepatocellular carcinoma, found an association between increased peripheral blood catecholamine levels and advanced clinical staging. These results indicate a dysfunction of the ANS in cancer patients with advanced clinical staging. Cancer patients may have nutritional deficits, whereas cancer-induced cachexia has been associated with modulation of the SNS [33,35].

Some clinical investigations with both cancer and healthy patients have shown an association between systemic catecholamine levels and psychological disorders [2,26,46]. The hypothesis of the present study was that the anxiety status could be correlated with plasma NE and E levels. However, only 14% of the total sample of the oral and oropharyngeal patients had high levels of anxiety, which were classified as moderate or severe. Our results showed no significant association between the anxiety scores on the BAI and the plasma catecholamine levels. These findings are consistent with previously published studies which found no significant association between catecholamine levels and global anxiety measurements in cancer patients [11,34,45]. However, a recent study found a significant positive correlation between anxiety scores and catecholamine levels in a sample of patients with liver cancer [22]. The low occurrence of high global anxiety levels in the HNSCC sample of the present study may have disrupted the intersection of hormonal and psychological data. Multiple regression analysis showed that the BAI subscale of “hand tremor” in the HSNCC group was an independent predictor for the plasma NE level, and that “heart pounding/racing” in the oropharyngeal SCC group was an independent predictor of the plasma E level. Although the present study did not analyze the clinical features associated to alcohol and tobacco withdrawal, the findings suggest a relationship between anxiety symptoms and alcohol and/or tobacco abstinence. For example, anxiety-related symptoms reported in the BAI, such as tachycardia and tremors, also are common symptoms found in alcohol withdrawal syndrome (AWS), after reduction or complete cessation of alcohol intake [47]. Since catecholamines derive from the SNS and the hyperactivity of this system is directly linked to AWS symptoms [37], a correlation between systemic catecholamines and the withdrawal symptomatology in HNSCC patients should be examined in further investigations. Other findings corroborate the results of the present study and have shown an association between psychological subscale measurements and catecholamine levels [2,26]. In addition, other symptoms and/or psychological disorders could be correlated with the modulation of catecholamine secretions in head and neck cancer patients. Depressive symptoms, mood changes, distress, social isolation, and fatigue have been shown to be possibly influenced by the SNS in cancer patients [2,26,46].

In contrast to what was observed in multiple regression models for the oral and oropharyngeal SCC groups, different independent variables predicting NE and E levels remained in the final model for the oral leukoplakia group. This data may be due to the absence of some variables, such as clinical staging, and the lower intensity of some relevant influencing factors for catecholamine levels, such as tobacco and alcohol consumption in the leukoplakia group. This is the first study exploring the relationship between sleep disturbances and catecholamine levels in HNSCC and oral leukoplakia patients. Sleep disruption and privation have been reported in cancer patients [34,48,49]. Carlson *et al*. [34] showed that breast cancer patients had worse sleep quality compared to healthy controls; however, sleep markers were not correlated with urinary NE and E levels. Another previous study found no association between plasma catecholamine and sleep deprivation in women with ovarian cancer [11]. In the findings of the present study, the worst sleep quality and sleep deprivation in the night before the blood collection were the only predicted biobehavioral variables for NE levels in the leukoplakia group, but they were not associated with hormonal concentrations in the HNSCC groups. Sleep disturbance has been linked to deregulated catecholamine secretion in non-cancer individuals [50–52]. For example, Mezick *et al*. [52] observed higher variability in sleep measurements among healthy patients with both high NE levels and high negative affect and anxiety. In our findings, multiple regression analysis defined higher anxiety scores as an independent psychological variable for E levels in the leukoplakia group. It is a fact that both sleep disturbance and elevated anxiety levels have been associated with systemic catecholamine levels in the oral leukoplakia patient group. This suggests that catecholamines could have a relevant role in bidirectional pathways involving psychological factors and sleep patterns.

The findings of the present study suggest that HNSCC patients display an SNS disturbance, which could have a relevant impact on disease progression. Although intratumoral catecholamine concentrations have not been measured, elevated hormonal levels in peripheral tissues, including the tumor microenvironment, may reflect those found in the blood. Increased concentrations of NE and/or E in the tumor microenvironment could induce tumor progression through adrenergic receptor activation, impacting HNSCC prognosis. Activation of β-adrenergic receptors by catecholamines increases the production of pro-inflammatory cytokines, chemokines, and factors affecting cancer progression [16–20]. In a previous study using an oral carcinogenesis model in rats, we demonstrated that increased pre-carcinogen NE concentrations in the normal tissue microenvironment were predictive for oral SCC occurrence, as well as lower expression of the tumor-suppressor gene pCDKN2a-p16. In addition, higher levels of NE in the tumor microenvironment were associated with lower immune response [53]. The presence of β-adrenergic receptors for NE and E has also been identified in HNSCC cell lines [18,54]. Increased levels of NE and E enhance the gene expression of VEGF, IL-6, and MMPs, molecules which are linked to tumor progression in HNSCC and other cancers [16–20,55]. High levels of catecholamines also could suppress the immune response against the cancer cells by reducing the activity of the cytotoxic T lymphocytes and NK cells [1,8–10,15]. Liu *et al*. [22] showed that plasma catecholamine levels were independent prognostic predictors of overall survival and tumor recurrence in hepatocellular carcinoma patients. Further studies are required to investigate the impact of systemic catecholamine levels on the HNSCC outcomes. The current study has limitations such as the relatively small sample of oropharyngeal SCC and oral leukoplakia patients and the absence of a group of healthy patients. Another limitation is not being able to assess whether the plasma NE and E levels have effects on prognostic factors of HNSCC survival, due to the patient’s short follow-up time period. Nevertheless, the present study is the first to report that plasma catecholamine levels are associated with biobehavioral variables and anxiety symptoms in HNSCC and oral leukoplakia patients.

In conclusion, our results reveal that patients with head and neck cancer display changes in circulating catecholamines. The hormone levels were associated with severity of alcohol consumption and specific anxiety symptoms, which are also commonly observed in the alcohol withdrawal status. Our findings have potential implications for clinical routine in head and neck cancer treatment. Deregulated secretion of epinephrine and norepinephrine could affect the tumor progression, a point that needs to be investigated in further studies. In addition, systemic catecholamines may become a useful physiological marker of hyperactivation of the sympathetic nervous system, induced by tobacco and alcohol withdrawal in patients with HNSCC. The assessment of these stress and anxiety-related endocrine responses can give a more detailed diagnosis of clinical impairment associated with chemical dependence and withdrawal in cancer patients. This could promote a more patient-specific approach during oncological treatment.
